# Racial and ethnic disparities in telehealth use before and after California's stay-at-home order

**DOI:** 10.3389/fpubh.2023.1222203

**Published:** 2023-08-22

**Authors:** Arturo Vargas Bustamante, Laura E. Martínez, Siavash Jalal, Nayelie Benitez Santos, Lucía Félix Beltrán, Jeremy Rich, Yohualli Balderas-Medina Anaya

**Affiliations:** ^1^Department of Health Policy and Management, Fielding School of Public Health, University of California, Los Angeles, Los Angeles, CA, United States; ^2^UCLA Latino Politics and Policy Institute, Los Angeles, CA, United States; ^3^Department of Obstetrics and Gynecology, David Geffen School of Medicine, University of California, Los Angeles, Los Angeles, CA, United States; ^4^Office of Advanced Research Computing, University of California, Los Angeles, Los Angeles, CA, United States; ^5^School of Nursing, University of California, San Francisco, San Francisco, CA, United States; ^6^HealthCare Partners Institute for Applied Research and Education, El Segundo, CA, United States; ^7^Department of Family Medicine and Community Health, UW-Madison School of Medicine and Public Health, Madison, WI, United States

**Keywords:** telehealth, disparities, chronic care management, COVID-19, race/ethnicity

## Abstract

**Introduction:**

Telehealth can potentially improve the quality of healthcare through increased access to primary care. While telehealth use increased during the COVID-19 pandemic, racial/ethnic disparities in the use of telemedicine persisted during this period. Little is known about the relationship between health coverage and patient race/ethnicity after the onset of the COVID-19 pandemic.

**Objective:**

This study examines how differences in patient race/ethnicity and health coverage are associated with the number of in-person vs. telehealth visits among patients with chronic conditions before and after California's stay-at-home order (SAHO) was issued on 19 March 2020.

**Methods:**

We used weekly patient visit data (in-person (*N* = 63, 491) and telehealth visits (*N* = 55, 472)) from seven primary care sites of an integrated, multi-specialty medical group in Los Angeles County that served a diverse patient population between January 2020 and December 2020 to examine differences in telehealth visits reported for Latino and non-Latino Asian, Black, and white patients with chronic conditions (type 2 diabetes, pre-diabetes, and hypertension). After adjusting for age and sex, we estimate differences by race/ethnicity and the type of insurance using an interrupted time series with a multivariate logistic regression model to study telehealth use by race/ethnicity and type of health coverage before and after the SAHO. A limitation of our research is the analysis of aggregated patient data, which limited the number of individual-level confounders in the regression analyses.

**Results:**

Our descriptive analysis shows that telehealth visits increased immediately after the SAHO for all race/ethnicity groups. Our adjusted analysis shows that the likelihood of having a telehealth visit was lower among uninsured patients and those with Medicaid or Medicare coverage compared to patients with private insurance. Latino and Asian patients had a lower probability of telehealth use compared with white patients.

**Discussion:**

To address access to chronic care management through telehealth, we suggest targeting efforts on uninsured adults and those with Medicare or Medicaid coverage, who may benefit from increased telehealth use to manage their chronic care.

## 1. Introduction

The rapid spread of SARS-CoV-2 resulted in the proclamation of a state of emergency in California by Governor Gavin Newson on 19 March 2020 (1). All individuals were ordered to stay at home or their place of residence, except those who were needed for the continuity of operations of federal critical infrastructure sectors. The healthcare delivery system in the State of California was restructured at that time to prioritize those at the highest risk and most vulnerable to COVID-19 (2, 3).

Telehealth is “the use of electronic information and telecommunication technologies to support long-distance clinical healthcare, patient and professional health-related education, public health, and health administration” and it includes video and audio visits (4). The use of telehealth after the issuance of California's stay-at-home order (SAHO) increased accessibility to primary care for patients with chronic conditions (5). Studies, however, show that telehealth use could have different effects on healthcare disparities across racial/ethnic groups (5–11). Other social determinants of health, such as age, income, socioeconomic status, and digital literacy can contribute to the widening of the digital divide that translates to inequitable healthcare access through telehealth (12). Those with inadequate broadband are often found in low-income and rural areas, which is an additional potential barrier (13, 14). Considering the impact of the COVID-19 pandemic on telehealth use for chronic care management and the potential risk of disparities in telehealth utilization across racial and ethnic groups, this is an important area of study, through which researchers can contribute toward reducing disparities.

Studies report that Black patients were more likely to seek and/or self-report telehealth use compared with white patients as an early onset response to the pandemic (15). The literature suggests that the reason behind the higher use of telehealth among Black individuals was their concern over contracting SARS-CoV-2 if they conducted in-person visits to seek care (15), supported by the fact that Black individuals were disproportionately impacted by COVID-19 (15, 16). Campos-Castillo and Anthony proposed that systemic racism contributed to health and healthcare disparities, likely raising awareness regarding the need for virtual care among Black individuals during the pandemic (15).

Shah et al. observed a significant increase in the number of telehealth visits by Latino and Black patients and a significant increase in the number of telehealth visits by patients with Medicare and Medicaid coverage (17). Others report significantly lower rates of telehealth use among patients of 65 years of age and older and telehealth utilization gaps between white and older Black and Latino patients (18, 19). Adepoju et al. reported disparities in telehealth use among Black, Asian, American Indian/Alaska Native, and Pacific Islander individuals as compared with white patients (20). Latinos were also less likely to use telehealth compared with non-Latinos (20).

Limited research has been conducted on the continuity of care among patients with chronic conditions. Existing studies have identified that higher care continuity was associated with telehealth use among patients with diabetes and hypertension during the pandemic; however, disparities exist in telehealth use by age, race, and income (21–24). Most research, however, has been conducted in resource-constrained health systems and community health centers. Little is known about the relationship between telehealth use and patient race/ethnicity and health coverage in other settings such as a commercial healthcare system. In this study, we used in-person and telehealth patient visit data obtained from seven primary care sites of an integrated, multi-specialty commercial healthcare system that serves a diverse patient population. This study investigates the associations between telehealth use and health coverage and the race/ethnicity of patients, managing their chronic conditions (type 2 diabetes, pre-diabetes, and hypertension) before and after the SAHO. These chronic conditions are three of the four topmost prevalent chronic conditions among US adults and carry significant health and economic costs in the United States (25). Thus, effective management of these diseases carries health and economic benefits.

## 2. Materials and methods

### 2.1. Data

We used de-identified data of patient visits from seven clinic sites for an integrated, multi-specialty medical group in Los Angeles County that serves a diverse patient population, primarily Latino, Black, Asian, and non-Latino white patients. Weekly in-person and telehealth visits from patients with the following characteristics were included in this study: 18 years of age and older, had one or more chronic conditions (type 2 diabetes, pre-diabetes, and hypertension), and had at least one primary care visit, either in-person or *via* telehealth, between 6 January 2020 and 21 December 2020.

### 2.2. Measures

We analyzed in-person and telehealth visits for two time periods: before (6 January 2020 to 16 March 2020) and after (23 March 2020 to 21 December 2020) the issuance of the SAHO (19 March 2020). In this study, telehealth visits include telephone and video visits, and patients might have had multiple in-person or telehealth visits each week. The main explanatory measures are race/ethnicity and type of health coverage. Race/ethnicity include the following categories: Latino, non-Latino Black, Asian (Asian and Pacific Islander were combined as one group), non-Latino white, and “Other” race/ethnicity (unspecified non-Latino and non-Latino American Indian or Alaska Natives). The types of health coverage analyzed include Medicare, Medicaid (or CHIP + Dual Eligible), private insurance, and those lacking health insurance (uninsured). We included sex (male or female) and age (four age groups: 18–39, 40–64, 65–75, and 76 years and older) as covariates in the regression models.

Since the number of patient visits was aggregated by week and primary care site, we first determined the percentage of in-person visits and telehealth visits for each week and each site. The accompanying Supplementary Table 1 summarizes the number and proportion of telehealth visits for each clinic site (Sites A to G) before and after the stay-at-home order. We then combined weekly visit data from all seven clinic sites to estimate the percentage of in-person and telehealth visits for each race/ethnicity and by health coverage before and after the SAHO. In total, we had 145,851 visits. Of the total visits, 80,849 were in-person and 65,002 were telehealth visits. In the analysis, we excluded race/ethnicity cases designated as “declined” or “NULL” (10,300; 7% of the total observations). We also excluded cases when the insurance type was not reported. Any missing data in our study were handled by listwise deletion since no other option was available as we had aggregated visit data. After this exclusion criteria, we had a total of 63,491 in-person visits and a total of 55,472 telehealth visits (18.4% of the total number of visits).

### 2.3. Logistic regression model

First, we investigated the impact of the SAHO on the percentage share of in-person vs. telehealth visits by health coverage and used a chi-square (χ^2^) test to assess statistical significance in the differences in telehealth use for each race/ethnicity and type of health coverage before and after the SAHO.

Second, we developed an interrupted time series logistic regression model to determine the effect of race/ethnicity and health coverage on telehealth utilization over the course of 51 weeks in 2020 (6 January 2020–21 December 2020). We used the weekly number of telehealth and in-person visits by health coverage for each site and by race/ethnicity.

We estimated the odds of telehealth use as the response variable at the visit level by the type of health coverage, race/ethnicity, and time (by week) as covariates. We included the date of the stay-at-home order of 19 March 2020 as a dummy variable in the model to examine the effect of exposure before and after the SAHO. Since the data were aggregated as weekly counts of in-person and telehealth visits, we were not able to determine demographic variables at the patient level. However, we used the percentage of female patient visits and the percentage of visits by patients 65 years and older (for each clinic site and for each week) to adjust for possible confounding of sex and age. Site-fixed effects were used to control the site dependency.

### 2.4. Two-way interactions

We included two-way interactions between some of the covariates in the model, and after a model selection process, we decided to keep the following interactions in our model:

1. Race/ethnicity with time and race/ethnicity before and after the SAHO;
2. Type of health coverage with time and type of health coverage before and after the SAHO;
3. Race/ethnicity with the type of health coverage;
4. Race/ethnicity with sex;
5. Race/ethnicity with age;
6. Type of health coverage with age;
7. Sex as a covariate before and after the SAHO;
8. Age as a covariate before and after the SAHO.

### 2.5. Statistical analysis

All statistical analyses were carried out using R (version 4.2.2) (26). The packages emmeans (27) and margins (28) were used to extract and summarize the results of the regression model. For statistical comparisons in average marginal effects, Bonferroni-corrected *p*-values are provided (Table 2). For statistical comparisons on two-way interaction terms, we conducted *z*-tests (Supplementary Table 2). An alpha level of 0.05 defined the statistical significance.

## 3. Results

Table 1 presents the chi-square test results comparing the percentage of in-person and telehealth visits for each race/ethnicity and by type of health coverage before and after the SAHO. Before the SAHO, the share of telehealth visits among patients with private insurance and for all clinic sites was higher than any other type of health coverage and in most race/ethnicity groups (Table 1). The proportion of telehealth visits by Black patients with Medicaid was slightly higher than the other types of health coverage (12.4% for Medicaid vs. 11.1% for private insurance) (Table 1). Our results also show that before the SAHO, patients with Medicare had a significant difference in the proportion of telehealth visits across the race/ethnicity groups (χ^2^ = 19.085, *df* = 4, *p* = 0.001), and a similar pattern was observed in the proportion of telehealth visits by race/ethnicity and the other types of health coverage (Table 1). After the SAHO, the proportion of telehealth visits was different among all race/ethnicity groups and for all types of health coverage: private (χ^2^= 57.314, *df* = 4, *p* < 0.001); Medicare (χ^2^= 415.834, *df* = 4, *p* < 0.001); Medicaid (χ^2^= 31.360, *df* = 4, *p* < 0.001); and uninsured (χ^2^= 82.990, *df* = 4, *p* < 0.001) (Table 1). In addition, the proportion of telehealth visits in Black patients with all types of health coverage was higher than any of the other race/ethnicity groups (Table 1).

**Table 1:** Percentage of in-person and telehealth visits for each race/ethnicity and by the type of health coverage before and after the SAHO.

	**Before stay-at-home order**				**After stay-at-home order**		
**Insurance Type**	**Race**	**Visit type**		**Total**	**Visit type**		**Total**
		**In-person**	**Telehealth**		**In-person**	**Telehealth**	
**Private**	White	1,541	196	1,737	1,968	3,605	5,573
		88.7 %	11.3 %		35.3 %	64.7 %	
	Latino	4,351	522	4,873	5,730	10,609	16,339
		89.3 %	10.7 %		35.1 %	64.9 %	
	Asian	559	64	623	860	1,217	2,077
		89.7 %	10.3 %		41.4 %	58.6 %	
	Black	656	82	738	818	1,834	2,652
		88.9 %	11.1 %		30.8 %	69.2 %	
	Other	437	52	489	628	1,144	1,772
		89.4 %	10.6 %		35.4 %	64.6 %	
	**Total**	7,544	916	8,460	10,004	18,409	28,413
		89.2 %	10.8 %		35.2 %	64.8 %	
**Medicare**	White	1,772	194	1,966	3,928	4,542	8,470
		90.1 %	9.9 %		46.4 %	53.6 %	
	Latino	6,549	620	7,169	14,229	15,811	30,040
		91.4 %	8.6 %		47.4 %	52.6 %	
	Asian	422	38	460	942	985	1,927
		91.7 %	8.3 %		48.9 %	51.1 %	
	Black	1,327	175	1,502	2,523	4,691	7,214
		88.3 %	11.7 %		35 %	65 %	
	Other	724	94	818	1,729	1,661	3,390
		88.5 %	11.5 %		51 %	49 %	
	***Total***	10,794	1,121	11,915	23,351	27,690	51,041
		90.6 %	9.4 %		45.7 %	54.3 %	
**Medicaid**	White	95	2	97	166	213	379
		97.9 %	2.1 %		43.8 %	56.2 %	
	Latino	519	42	561	1068	1324	2392
		92.5 %	7.5 %	100 %	44.6 %	55.4 %	
	Asian	15	1	16	44	31	75
		93.8 %	6.2 %		58.7 %	41.3 %	
	Black	99	14	113	205	397	602
		87.6 %	12.4 %		34.1 %	65.9 %	
	Other	52	2	54	82	129	211
		96.3 %	3.7 %		38.9 %	61.1 %	
	***Total***	780	61	841	1,565	2,094	3,659
		92.7 %	7.3 %		42.8 %	57.2 %	
	Fisher's *p* = 0.040						
**Uninsured**	White	643	9	652	1,375	1,077	2,452
		98.6 %	1.4 %		56.1 %	43.9 %	
	Latino	1,431	38	1,469	3,812	2,604	6,416
		97.4 %	2.6 %		59.4 %	40.6 %	
	Asian	190	3	193	425	377	802
		98.4 %	1.6 %		53 %	47 %	
	Black	316	8	324	601	701	1,302
		97.5 %	2.5 %		46.2 %	53.8 %	
	Other	192	5	197	470	359	829
		97.5 %	2.5 %		56.7 %	43.3 %	
	***Total***	2,772	63	2,835	6,683	5,118	11,801
		97.8 %	2.2 %		56.6 %	43.4 %	
	Fisher's *p* = 0.458						

**Figure 1:**
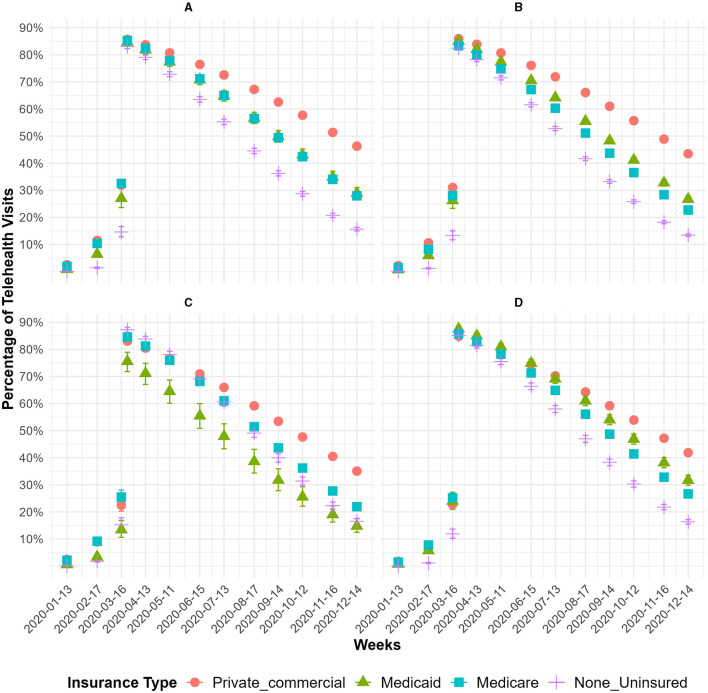
The monthly percentage of telehealth visits over time for each race/ethnicity and by insurance type between January 2020 and December 2020. **(A)** White. **(B)** Latino. **(C)** Asian. **(D)** Black.

To determine the effect of race/ethnicity and health coverage on telehealth utilization, we developed an interrupted time series logistic regression model. Figure 1 shows the predicted probability from the regression model summarized in Section 2.3. At the beginning of 2020, we observed that the percentage of telehealth visits ranged from 0.03% to 7.2% (Figures 1A–D). The uninsured had the lowest percentage of telehealth visits and those with private insurance had the highest percentage of telehealth visits. Right before the SAHO, the percentage of telehealth visits increased from 10.6% to 32.5% (Figures 1A–D). White patients with private insurance or Medicare showed the highest percentage share of telehealth visits.

Right after the SAHO, we observed a shift in telehealth visits with a range of 75.5%−87.5% (Figures 1A–D), where the highest percentage share of telehealth visits was among Black patients with Medicaid, and for most of the other race/ethnicities and type of health coverage, it was above 83%. After the SAHO, the percentage share of telehealth visits decreased, and at the end of 2020, telehealth use ranged from 12.4% to 50.6% (Figures 1A–D). At the end of 2020, the average share of telehealth visits by patients with private insurance was 42.4%, and for Medicare, it was 24.3%, Medicaid 27.2%, and those uninsured 14.9%. Before the SAHO, we observed that, on average, for each week, the odds of telehealth use increased by 48.5%, and after the SAHO, the odds of telehealth use decreased by 36% (data not shown but available upon request).

We did not observe a significant difference between the increase in telehealth use before the SAHO and the decline of telehealth use after the SAHO for white, Latino, Asian, and Black patients (Supplementary Table 2). The interaction between race/ethnicity and type of health coverage indicated that Latino patients with Medicare had an additional lower odds ratio compared with Latino patients with private insurance (OR = 0.85, 95% CI [0.78, 0.92], *p* < 0.001) (Supplementary Table 2). In addition, Asian patients without insurance had higher odds of telehealth use compared with uninsured white patients (OR = 1.7, 95% CI [1.38, 2.10], *p* < 0.001). All results of the interaction effects are available in Supplementary Table 2.

Right before the SAHO, the percentage of female patients was positively associated with the share of telehealth visits for all races/ethnicities, with a weaker association for Latino patients (OR = 0.83, 95% CI [0.78, 0.89], *p* < 0.001) (Supplementary Table 2). Right after the SAHO, the percentage of female patients to the share of telehealth visits by all races/ethnicities was also positive. Immediately before and after the SAHO, the percentage of patients aged 65 years and older was negatively associated with the share of telehealth use for all races/ethnicities, with a more pronounced negative effect on Latino patients. Moreover, this negative effect on the percentage of patients aged 65 years and older was consistent before and after the SAHO for all types of health coverage (Supplementary Table 2).

Table 2 shows the average marginal effect (AME) of each predictor as the probability of telehealth visits at different time periods of 2020 as follows: 2 months before the SAHO, right after the SAHO, and 3 months after the SAHO. Both the percentage of female patients and the percentage of patients aged 65 years and older were centered at the mean and operationalized as 10% increments. We included the time before and after as two covariates. The coefficient of time before and after represents the effect of the SAHO timing on the odds of telehealth use, respectively. The coefficient of the stay-at-home order [after] is interpreted as the immediate change in the odds of telehealth use after the SAHO was implemented. We observed a consistent negative effect in the percentage of patients aged 65 years and older; thus, the proportion of telehealth visits decreased on average (January 2020, AME = −0.002, 95% CI [−0.003, −0.001], and December 2020, AME = −0.015, 95% CI [−0.019, −0.012], *p* < 0.01) (Table 2). When comparing uninsured patient visits with private insurance, we found that, on average, the proportion of telehealth visits decreased toward the end of 2020 (January 2020, AME = −0.039, 95% CI [−0.048, −0.030], *p* < 0.001, and December 2020, AME = −0.273, 95% CI [−0.290, −0.256], *p* < 0.001) (Table 2). By the end of 2020, the share of telehealth visits conducted by patients with Medicaid (December 2020, AME = −0.147, 95% CI [−0.181, −0.113], *p* < 0.001) and Medicare (December 2020, AME = −0.176, 95% CI [−0.191, −0.161], *p* < 0.001) was less than those with private insurance. Furthermore, the average predicted probability of telehealth visits for all race/ethnicity categories between January and December of 2020 was not substantially different, except for a decrease in the probability of visits by Asian patients during September (AME = −0.071, 95% CI [−0.105, −0.037], *p* < 0.01) and December 2020 (AME = −0.076, 95% CI [−0.106, −0.046], *p* < 0.001). Table 2 also summarizes the results of telehealth visits conducted for each clinic site relative to site A, which was used as the reference.

**Table 2:** Summary of the average marginal effect (AME) of covariates used in the model to evaluate the probability of telehealth visits.

	**Before stay-at-home order**			**After stay-at-home order**			
	**Average marginal effect (95% CI)**			**Average marginal effect (95% CI)**			
**Variable**	January 2020	February 2020	March 2020 (before the stay-at-home order)	March 2020 (after the stay-at-home order)	June 2020	September 2020	December 2020
**Percentage of patients of 65 years of age and older** ^**b**^	−0.002	−0.008^***a^	−0.020^***^	−0.011^***^	−0.016^***^	−0.019^***^	−0.015^***^
	(−0.003, −0.001)	(−0.011, −0.005)	(−0.028, −0.013)	(−0.014, −0.008)	(−0.021, −0.012)	(−0.024, −0.014)	(−0.019, −0.012)
**Percentage of female patients** ^**b**^	0.004^***^	0.014^***^	0.034^***^	0	0.0002	0.0008	0.0009
	(0.002, 0.006)	(0.009, 0.019)	(0.023, 0.046)	(−0.003, 0.003)	(−0.005, 0.005)	(−0.005, 0.006)	(−0.004, 0.005)
**Health coverage (ref** = **private)**							
**Medicaid**	−0.024	−0.046^*^	−0.033	−0.006	−0.050	−0.111^***^	−0.147^***^
	(−0.039, −0.010)	(−0.070, −0.022)	(−0.106, 0.041)	(−0.036, 0.023)	(−0.083, −0.018)	(−0.142, −0.079)	(−0.181, −0.113)
**Medicare**	−0.011	−0.014	−0.0004	−0.008	−0.059^***^	−0.132^***^	−0.176^***^
	(−0.020, −0.003)	(−0.026, −0.003)	(−0.029, 0.028)	(−0.018, 0.003)	(−0.070, −0.048)	(−0.143, −0.121)	(−0.191, −0.161)
**Uninsured**	−0.039^***^	−0.104^***^	−0.138^***^	−0.011	−0.103^***^	−0.226^***^	−0.273^***^
	(−0.048, −0.030)	(−0.116, −0.092)	(−0.185, −0.090)	(−0.027, 0.005)	(−0.120, −0.087)	(−0.241, −0.210)	(−0.290, −0.256)
**Race/ethnicity (ref** = **white)**							
**Asian**	−0.0006	−0.019	−0.074	−0.020	−0.045	−0.071^**^	−0.076^***^
	(−0.010, 0.008)	(−0.036, −0.001)	(−0.134, −0.013)	(−0.051, 0.011)	(−0.080, −0.009)	(−0.105, −0.037)	(−0.106, −0.046)
**Black**	−0.003	−0.018	−0.051	0.010	0.011	0.005	−0.004
	(−0.010, 0.003)	(−0.032, −0.003)	(−0.098, −0.005)	(−0.008, 0.028)	(−0.010, 0.032)	(−0.018, 0.028)	(−0.029, 0.020)
**Latino**	−0.002	−0.006	−0.014	−0.016	−0.026	−0.029	−0.023
	(−0.007, 0.004)	(−0.018, 0.006)	(−0.051, 0.022)	(−0.030, −0.002)	(−0.042, −0.010)	(−0.046, −0.012)	(−0.041, −0.005)
**“Other” race**	0.033^*^	0.03	−0.054	−0.038	−0.023	0.015	0.048
	(0.016, 0.051)	(0.009, 0.051)	(−0.112, 0.003)	(−0.062, −0.014)	(−0.048, 0.003)	(−0.012, 0.042)	(0.017, 0.079)
**Stay-at-home order placed**			0.601^***^	0.602^***^			
			(0.575, 0.626)	(0.578, 0.627)			
**Time before stay-at-home order** ^**c**^	0.0053^***^	0.0219^***^	0.0614^***^				
	(0.005, 0.006)	(0.019, 0.024)	(0.050, 0.073)				
**Time after stay-at-home order** ^**c**^				−0.0627^***^	−0.0957^***^	−0.1054^***^	−0.0806^***^
				(−0.070, −0.055)	(−0.107, −0.085)	(−0.117, −0.094)	(−0.089, −0.072)
**Site (ref** = **site A)**							
**Site B**	−0.014^***^	−0.047^***^	−0.106^***^	−0.062^***^	−0.102^***^	−0.130^***^	−0.115^***^
	(−0.018, −0.010)	(−0.055, −0.039)	(−0.123, −0.088)	(−0.072, −0.052)	(−0.118, −0.087)	(−0.149, −0.111)	(−0.133, −0.098)
**Site C**	−0.003	−0.009	−0.018	−0.008	−0.014	−0.020	−0.019
	(−0.005, −0.001)	(−0.015, −0.002)	(−0.031, −0.005)	(−0.014, −0.002)	(−0.024, −0.004)	(−0.034, −0.006)	(−0.033, −0.005)
**Site D**	−0.020^***^	−0.069^***^	−0.162^***^	−0.1173^***^	−0.1829^***^	−0.2156^***^	−0.1802^***^
	(−0.025, −0.015)	(−0.077, −0.060)	(−0.180, −0.145)	(−0.126, −0.108)	(−0.195, −0.171)	(−0.231, −0.200)	(−0.195, −0.165)
**Site E**	−0.022^***^	−0.075^***^	−0.1799^***^	−0.1399^***^	−0.2134^***^	−0.2449^***^	−0.2007^***^
	(−0.027, −0.017)	(−0.084, −0.066)	(−0.2004, −0.1595)	(−0.156, −0.124)	(−0.234, −0.193)	(−0.267, −0.223)	(−0.219, −0.183)
**Site F**	−0.017^***^	−0.056^***^	−0.130^***^	−0.0824^***^	−0.1333^***^	−0.1644^***^	−0.1422^***^
	(−0.021, −0.013)	(−0.064, −0.048)	(−0.147, −0.113)	(−0.091, −0.073)	(−0.147, −0.120)	(−0.182, −0.147)	(−0.158, −0.126)
**Site G**	−0.017^***^	−0.058^***^	−0.134^***^	−0.0866^***^	−0.1394^***^	−0.1709^***^	−0.1472^***^
	(−0.021, −0.013)	(−0.067, −0.049)	(−0.153, −0.115)	(−0.099, −0.074)	(−0.158, −0.121)	(−0.192, −0.150)	(−0.166, −0.129)

^a***^p < 0.001; ^**^p < 0.01; ^*^p < 0.05.

Asterisks specify degrees of significance as adjusted for multiple comparisons. Bonferroni-corrected p-values are shown., ^b^percentage increment by 10%., ^c^weekly increments.

To investigate the effect of language on telehealth use, we used a separate logistic regression model. This model included language as a categorical variable with an interaction term between language and race/ethnicity. Since patient visit data were aggregated, we were unable to incorporate health coverage into this model. In addition, we had limited data points for certain race/ethnicity groups and language combinations; thus, we categorized language as either English or non-English speakers. For consistency with the original model, except for the type of health insurance, we included other covariates and interaction terms presented in Section 2.4.

Using a likelihood ratio test, we evaluated the overall impact of the language and race/ethnicity interaction, which indicated that language did not contribute to the effect of race/ethnicity on telehealth use (χ^2^ = 4.9992, *df* = 4, *p* = 0.2874). Moreover, the main effect of language being non-zero was compatible with the data. The result showed that non-English speakers had approximately 22% lower odds of utilizing telehealth while holding all other variables constant (OR = 0.783, *z* = −13.620, *p* < 0.001).

## 4. Discussion

One of our study's main strengths is that the sample includes a diverse number of patient visits from the main race/ethnic groups in California. However, since our study was conducted in a specific commercial group, it is not fully representative of the state's population or of individuals who receive care at other health systems, which limits the generalizability of our findings. Another strength is that we illustrate telehealth utilization across different categories of race/ethnicity and insurance in a commercial setting, while previous studies only provided findings on resource-scarce settings.

A limitation of this study is the use of aggregate patient visit data rather than individual patient-level data. The use of aggregate patient visit data limits the ability to determine patient-level associations with clinical demographics and/or other variables, such as age, gender, and language use at each primary care visit. However, we were able to operationalize age and gender as a proportion at the clinic level. Although individual patient-level data are preferable and are more flexible for statistical analysis, aggregated data have been shown to be appropriate for statistical analysis when patient-level data are unavailable (29, 30). Moreover, this study did not address other potential barriers that underrepresented minority groups could experience while accessing virtual care, such as limited access to technology-enabled resources faced during the pandemic (5, 8, 11). In this study, we were unable to disaggregate access to telehealth visits due to COVID-19 or other health conditions. Additional limitations of our study are the potential for selection bias and unmeasured factors such as socioeconomic status and other confounders.

In this study, we sought to examine the relationship between patient race/ethnicity and health coverage, with telehealth visits among patients with chronic conditions before and after California's SAHO, issued on 19 March 2020. Our results confirm that as the norm was across primary care in the US prior to the pandemic, most visits by patients with chronic conditions were conducted in person before the SAHO, and there was an immediate shift to telehealth use once the SAHO was enacted. Thus, our findings are consistent with the existing literature on telehealth, accounting for a low percentage of healthcare delivery before the COVID-19 pandemic (5, 8, 11). When examining telehealth use by race/ethnicity, we found that telehealth visits by Black patients exceeded visits by white patients, irrespective of the type of health insurance coverage. This finding supports the results from previous findings, where interviewees perceived that Black and white patient populations experienced fewer technology hurdles with telehealth use (5).

Our findings also point out that patient visits by Latino and non-Latino Black, Asian, or white patients with private insurance had the highest share of telehealth visits when compared with patients with Medicaid or Medicare and those uninsured. Collectively, Asian patients with Medicaid coverage had the lowest percentage of telehealth use compared with Latino, Black, or white patients. Other studies have found that Asian patients adapted well to technology and have higher rates of video visits compared with Black and Latino patients (31). One study found that East and Southeast Asian patients specifically had overall lower telehealth utilization rates compared with non-Latino whites (32). Additionally, uninsured individuals (compared with those with private insurance) and those with limited broadband coverage engaged less with telehealth during the pandemic (32).

Our findings suggest that telehealth access barriers exist for uninsured Latino, Black, and white patients and Asian and Pacific Islander patients with Medicaid coverage. Factors that can account for these disparities could be discomfort or lack of familiarity with telehealth, digital literacy, limited English proficiency, access to devices and broadband, and barriers to healthcare access that are a result of income or low socioeconomic status (12, 33–35). While previous research on this topic has been conducted within safety net clinics, we also found differences in access to telehealth services based on race/ethnicity and the type of health coverage in a commercial healthcare system. This study also shows that even though patients are receiving care from a well-resourced and integrated healthcare system, race/ethnicity disparities in telehealth use for the continuity of primary care are present.

Immediately following California's SAHO, telehealth became a prominent mode of delivery of primary care services for patients living with chronic conditions. Future telehealth state and federal legislation can address gaps in telehealth use by race/ethnicity and by health coverage type. The expansion of telehealth made it possible for millions of adults with chronic conditions to have continuity of healthcare during a pandemic. Recently, Congress expanded funding and telehealth flexibilities, and permanent changes were made through 31 December 2024, continuing to allow Medicare patients to use telehealth services without geographic limitations (2). Future research should investigate the evolution of telehealth use by race/ethnicity and health insurance coverage after more in-person activities resumed starting in 2021 to inform the future funding and service model of telehealth services.

Our study shows that telehealth visits increased after California's stay-at-home order was issued. However, the likelihood of telehealth utilization decreased over time by the end of 2020. Our findings also highlight the gaps in telehealth use among Latino, Black, and Asian patients compared with white patients. Our results from evaluating differences in health coverage show that patients with Medicaid or Medicare, and those uninsured, consistently showed lower telehealth use compared with patients with private health insurance coverage. We also confirmed that non-English speakers were less likely to conduct visits *via* telehealth. However, language was not an additional barrier to the effect of race/ethnicity and vice versa.

Future research should investigate how to increase telehealth access to underrepresented minority and underserved patient populations, particularly among those who are uninsured or have limited health coverage. Future research should also determine barriers associated with telehealth visits, especially for patients with Limited English Proficiency (LEP) and with multiple comorbidities, and evaluate the impact of shifting to telehealth on continuity of care, especially among individuals experiencing a cultural-linguistic divide and/or challenges with technology literacy (i.e., older adults; patients requiring American Sign Language interpreters).

## Data availability statement

The original contributions presented in the study are included in the article/Supplementary material, further inquiries can be directed to the corresponding author.

## Ethics statement

No human studies are presented in the manuscript. No potentially identifiable images or data are presented in this study.

## Author contributions

AB, LM, and SJ contributed to the conception and design of the study. LM, SJ, and NB performed the statistical analysis. AB and LM wrote the first draft of the manuscript. AB, LM, SJ, and YA wrote the revised version of this manuscript. All authors contributed to the manuscript revision, read, and approved the submitted version.
