# Supplementary material for: Racial and ethnic disparities in telehealth use before and after California's stay-at-home order
Source: Front Public Health. 2023 Aug 22;11:1222203. doi: 10.3389/fpubh.2023.1222203 (PMC10477577; doi:10.3389/fpubh.2023.1222203)
Supplement: Supplementary file 1 [file Table_1.DOCX]

Supplementary Material

Racial and ethnic disparities in telehealth use before and after California’s stay-at-home order

# Supplementary Table 1. Please see the accompanying excel that includes this supplementary material.

# Supplementary Table 2. Adjusted odds ratios from interrupted time series of race/ethnicity on telehealth visits.

| **Variable** | **Odds Ratios** | **Standard Error** | **95% CI** | ***p-*value** |
| --- | --- | --- | --- | --- |
| Site A | 0.02 | 0.01 | 0.02 – 0.04 | <0.001 |
| Site B | 0.01 | 0 | 0.01 – 0.02 | <0.001 |
| Site C | 0.02 | 0 | 0.01 – 0.03 | <0.001 |
| Site D | 0.01 | 0 | 0.01 – 0.01 | <0.001 |
| Site E | 0.01 | 0 | 0.01 – 0.01 | <0.001 |
| Site F | 0.01 | 0 | 0.01 – 0.02 | <0.001 |
| Site G | 0.01 | 0 | 0.01 – 0.02 | <0.001 |
| Latino | 0.85 | 0.19 | 0.55 – 1.32 | 0.457 |
| Asian | 1.09 | 0.43 | 0.49 – 2.29 | 0.833 |
| Black | 0.7 | 0.21 | 0.38 – 1.27 | 0.241 |
| Other | 4.95 | 1.51 | 2.71 – 8.94 | <0.001 |
| SAHO [after] | 13.36 | 1.39 | 10.90 – 16.41 | <0.001 |
| Time (in weeks) before | 1.38 | 0.03 | 1.32 – 1.45 | <0.001 |
| Time (in weeks) after | 0.69 | 0.02 | 0.66 – 0.72 | <0.001 |
| Medicaid | 0.27 | 0.15 | 0.08 – 0.73 | 0.016 |
| Medicare | 0.7 | 0.12 | 0.51 – 0.98 | 0.037 |
| Uninsured | 0.01 | 0.01 | 0.00 – 0.05 | <0.001 |
|  |  |  |  |  |
| Female | 1.28 | 0.05 | 1.18 – 1.39 | <0.001 |
| Over 65 years | 0.89 | 0.02 | 0.84 – 0.94 | <0.001 |
| Race [Latino] * SAHO [after] | 1.08 | 0.12 | 0.87 – 1.33 | 0.506 |
|  |  |  |  |  |
| Race [Asian] * SAHO [after] | 1.34 | 0.27 | 0.90 – 2.01 | 0.151 |
|  |  |  |  |  |
| Race [Black] * SAHO [after] | 1.49 | 0.22 | 1.11 – 2.00 | 0.007 |
|  |  |  |  |  |
| Race [Other] * SAHO [after] | 1.03 | 0.19 | 0.72 – 1.48 | 0.87 |
|  |  |  |  |  |
| Race [Latino] * time before | 1.01 | 0.03 | 0.96 – 1.06 | 0.671 |
|  |  |  |  |  |
| Race [Asian] * time before | 0.95 | 0.04 | 0.87 – 1.04 | 0.262 |
|  |  |  |  |  |
| Race [Black] * time before | 0.99 | 0.03 | 0.92 – 1.06 | 0.77 |
|  |  |  |  |  |
| Race [Other] * time before | 0.84 | 0.03 | 0.78 – 0.90 | <0.001 |
|  |  |  |  |  |
| Race [Latino] * time (after March 20, 2020) | 0.99 | 0.03 | 0.94 – 1.04 | 0.573 |
|  |  |  |  |  |
| Race [Asian] * time (after March 20, 2020) | 1.05 | 0.05 | 0.95 – 1.14 | 0.334 |
| Race [Black] * time (after March 20, 2020) | 1.01 | 0.04 | 0.94 – 1.08 | 0.834 |
| Race [Other} * time (after March 20, 2020) | 1.21 | 0.05 | 1.12 – 1.30 | <0.001 |
| Race [Latino] * Medicaid | 1 | 0.12 | 0.79 – 1.27 | 0.975 |
|  |  |  |  |  |
| Race [Asian] * Medicaid | 0.68 | 0.19 | 0.39 – 1.17 | 0.165 |
|  |  |  |  |  |
| Race [Black] * Medicaid | 1.36 | 0.2 | 1.02 – 1.83 | 0.039 |
|  |  |  |  |  |
| Race [Other] * Medicaid | 1.34 | 0.25 | 0.93 – 1.94 | 0.123 |
|  |  |  |  |  |
| Race [Latino] * Medicare | 0.85 | 0.04 | 0.78 – 0.92 | <0.001 |
|  |  |  |  |  |
| Race [Asian] * Medicare | 1.16 | 0.09 | 0.99 – 1.34 | 0.061 |
|  |  |  |  |  |
| Race [Black] * Medicare | 1.12 | 0.07 | 0.99 – 1.27 | 0.068 |
|  |  |  |  |  |
| Race [Other] * Medicare | 0.85 | 0.06 | 0.74 – 0.97 | 0.019 |
|  |  |  |  |  |
| Race [Latino] * Uninsured | 0.94 | 0.06 | 0.83 – 1.06 | 0.298 |
|  |  |  |  |  |
| Race [Asian] * Uninsured | 1.7 | 0.18 | 1.38 – 2.10 | <0.001 |
|  |  |  |  |  |
| Race [Black] * Uninsured | 1.26 | 0.11 | 1.05 – 1.50 | 0.012 |
|  |  |  |  |  |
| Race [Other] * Uninsured | 0.95 | 0.1 | 0.78 – 1.17 | 0.657 |
|  |  |  |  |  |
| SAHO [after] * Medicaid | 1.2 | 0.27 | 0.77 – 1.89 | 0.425 |
|  |  |  |  |  |
| SAHO [after] * Medicare | 0.97 | 0.08 | 0.82 – 1.14 | 0.671 |
|  |  |  |  |  |
| SAHO [after] * Uninsured | 2.34 | 0.47 | 1.59 – 3.49 | <0.001 |
|  |  |  |  |  |
|  |  |  |  |  |
| time before * Medicaid | 1.1 | 0.07 | 0.98 – 1.25 | 0.107 |
|  |  |  |  |  |
| time before * Medicare | 1.03 | 0.02 | 1.00 – 1.07 | 0.08 |
|  |  |  |  |  |
| time before * Uninsured | 1.35 | 0.11 | 1.17 – 1.60 | <0.001 |
|  |  |  |  |  |
| time after * Medicaid | 0.89 | 0.05 | 0.78 – 1.00 | 0.058 |
|  |  |  |  |  |
| time after * Medicare | 0.95 | 0.02 | 0.91 – 0.98 | 0.005 |
|  |  |  |  |  |
| time after * Uninsured | 0.71 | 0.06 | 0.60 – 0.83 | <0.001 |
|  |  |  |  |  |
| Race [Latino] * Female | 0.83 | 0.03 | 0.78 – 0.89 | <0.001 |
|  |  |  |  |  |
| Race [Asian] * Female | 0.98 | 0.04 | 0.91 – 1.05 | 0.581 |
| Race [Black] * Female | 1.03 | 0.04 | 0.96 – 1.11 | 0.36 |
| Race [Other] * Female | 0.96 | 0.03 | 0.89 – 1.03 | 0.226 |
| Race [Latino] * Over 65 years | 0.8 | 0.02 | 0.76 – 0.83 | <0.001 |
|  |  |  |  |  |
| Race [Asian] * Over 65 years | 1.04 | 0.03 | 0.99 – 1.10 | 0.148 |
|  |  |  |  |  |
| Race [Black] * Over 65 years | 0.96 | 0.02 | 0.92 – 1.01 | 0.135 |
|  |  |  |  |  |
| Race [Other] * Over 65 years | 1.08 | 0.03 | 1.03 – 1.13 | 0.003 |
|  |  |  |  |  |
| SAHO [after] × c p  female | 0.82 | 0.03 | 0.76 – 0.88 | <0.001 |
|  |  |  |  |  |
| Medicaid * Over 65 years of age | 1.04 | 0.03 | 0.98 – 1.11 | 0.176 |
|  |  |  |  |  |
| Medicare * Over 65 years of age | 1.03 | 0.01 | 1.01 – 1.06 | 0.018 |
|  |  |  |  |  |
| Uninsured * Over 65 years of age | 1.07 | 0.02 | 1.03 – 1.11 | 0.001 |
|  |  |  |  |  |
|  |  |  |  |  |
| SAHO [after] × Over 65 years of age | 1.04 | 0.02 | 0.99 – 1.08 | 0.121 |
|  |  |  |  |  |
| **Number of Observations** | **5,712** | | | |
